# Machine Learning-Based Prediction of Loneliness in Older Adults With Cognitive Decline

**DOI:** 10.1093/geroni/igaf122.3115

**Published:** 2025-12-31

**Authors:** Bada Kang, Min Kyung Park, Dahye Hong, Sion Kim, Minji Kang, Haneul Oh

**Affiliations:** Yonsei University College of Nursing, Seoul, Republic of Korea; Yonsei University College of Nursing, Seoul, Republic of Korea; Yonsei University College of Nursing, Seoul, Republic of Korea; Yonsei University College of Nursing, Seoul, Republic of Korea; Yonsei University College of Nursing, Seoul, Republic of Korea; Yonsei University College of Nursing, Seoul, Republic of Korea

## Abstract

Loneliness, a significant public health concern—particularly among older adults—is associated with several adverse health outcomes. Individuals with mild cognitive impairment (MCI) and subjective cognitive decline (SCD) are more likely to experience loneliness owing to reduced social engagement and poor emotional regulation. Because previous research has primarily focused on cross-sectional associations between health-related factors and loneliness levels, the predictors of future loneliness in this population remain largely unexplored. Using a two-year longitudinal dataset, this study aimed to develop machine learning-based predictive models to identify factors associated with loneliness levels one year after baseline assessment in older adults with MCI and SCD. A total of 78 older adults (n = 56 SCD and 22 MCI) completed assessments at two time points. In the first year, baseline demographic and health-related data were collected through surveys, while sleep and physical activity data were obtained over two weeks via a 24-hour wrist-worn Actiwatch. Twelve months later, real-time loneliness levels were measured over two weeks employing a mobile Ecological Momentary Assessment approach. Among the tested machine learning models, the Random Forest model demonstrated the highest predictive performance for loneliness one year later (area under the receiver operating characteristic curve [AUC] = 0.970). Sedentary behavior emerged as the strongest predictor, followed by physical movement. These findings highlight the potential of machine learning to predict loneliness in older adults at risk of dementia and emphasize the importance of maintaining adequate physical activity to mitigate loneliness in this vulnerable population.

